# Association between immunosuppressants and poor antibody responses to SARS-CoV-2 vaccines in patients with autoimmune liver diseases

**DOI:** 10.3389/fimmu.2022.988004

**Published:** 2022-10-05

**Authors:** Hu Li, Yuting Wang, Ling Ao, Mingxia Ke, Zhiwei Chen, Min Chen, Mingli Peng, Ning Ling, Peng Hu, Dachuan Cai, Dazhi Zhang, Hong Ren

**Affiliations:** Department of Infectious Diseases, Key Laboratory of Molecular Biology for Infectious Diseases (Ministry of Education), Institute for Viral Hepatitis, The Second Affiliated Hospital, Chongqing Medical University, Chongqing, China

**Keywords:** severe acute respiratory syndrome coronavirus 2, coronavirus disease 2019, vaccine, immunogenicity, safety, autoimmune liver diseases

## Abstract

The antibody and B cell responses after inactivated SARS-CoV-2 vaccination have not been well documented in patients with autoimmune liver disease (AILD). Therefore, we conducted a prospective observational study that included AILD patients and healthy participants as controls between July 1, 2021, and September 30, 2021, at the Second Affiliated Hospital of Chongqing Medical University. All adverse events (AEs) after the COVID-19 vaccination were recorded and graded. Immunoglobulin (Ig)-G antibodies against the receptor-binding domain (RBD) of the SARS-CoV-2 spike protein (anti-RBD-IgG) and neutralizicadng antibodies (NAbs) were tested following full-course vaccination (BBIBP-CorV or CoronaVac). In addition, SARS-CoV-2-specific B cells were detected by flow cytometry. In total, 76 AILD patients and 136 healthy controls (HCs) were included. All AEs were mild and self-limiting, and the incidences were similar between the AILD and HCs. The seropositivity rates of anti-RBD-IgG and NAbs in AILD were 97.4% (100% in HCs, p = 0.13) and 63.2% (84.6% in HCs, p < 0.001), respectively. The titers of anti-RBD-IgG and NAbs were significantly lower in AILD patients than those in HCs. After adjusting for confounders, immunosuppressive therapy was an independent risk factor for low-level anti-RBD-IgG (adjusted odds ratio [aOR]: 4.7; 95% confidence interval [CI], 1.5-15.2; p = 0.01) and a reduced probability of NAbs seropositivity (aOR, 3.0; 95% CI, 1.0-8.9; p = 0.04) in AILD patients. However, regardless of immunosuppressants, the SARS-CoV-2-specific memory B cells responses were comparable between the AILD and HC groups. Our results suggest that inactivated SARS-CoV-2 vaccines (BBIBP-CorV and CoronaVac) are safe, but their immunogenicity is compromised in patients with AILD. Moreover, immunosuppressants are significantly associated with poor antibody responses to the SARS-CoV-2 vaccines. These results could inform physicians and policymakers about decisions on screening the populations at higher risk of poor antibody responses to SARS-CoV-2 vaccines and providing additional vaccinations in patients with AILD.

## Introduction

Coronavirus disease 2019 (COVID-19), caused by severe acute respiratory syndrome coronavirus 2 (SARS-CoV-2) infection, has become a significant global public health threat. To date, more than 528 million people have been diagnosed with COVID-19, and more than 6 million deaths have been confirmed worldwide (1). Numerous studies have shown that people with comorbidities, including chronic liver disease, are highly vulnerable and have worse outcomes from COVID-19 than those without underlying liver disease (2–5). Therefore, liver societies have recommended vaccination against SARS-CoV-2 for patients with chronic liver diseases (6, 7). However, a series of case reports suggest that mRNA-based vaccines may induce autoimmune liver disease (8–16). This has caused concern among hepatologists as well as patients with autoimmune liver diseases (AILD) (17, 18).

AILD are chronic immune-mediated liver diseases that includ a wide range of disorders, such as autoimmune hepatitis (AIH), primary biliary cholangitis (PBC) and AIH-PBC overlap syndrome, which are frequently treated with either broad or targeted immunosuppressants. Moreover, some AILD patients usually have other autoimmune diseases, such as rheumatoid arthritis, systemic lupus erythematosus and Sjögren’s syndrome, which also require lifelong immunosuppressive drug therapy. Existing data have shown that immunosuppressants can significantly reduce the antibody response to COVID-19 mRNA vaccines or the Johnson & Johnson vaccine in liver transplant patients (19) and immune-mediated inflammatory disorders (20, 21).

In China, inactivated vaccines (BBIBP-CorV or CoronaVac) are widely used COVID-19 vaccines. Systematic evaluation of the safety and immunogenicity of these vaccines in people with AILD has been rare. Here, we aim to evaluate the safety and antibody responses after the whole-course COVID-19 vaccination and explore the association between immunosuppressants and antibody responses to inactivated SARS-CoV-2 vaccines in patients with AILD.

## Patients and methods

### Study design and participants

Between July 1, 2021, and September 30, 2021, we performed a prospective observational study at the Second Affiliated Hospital of Chongqing Medical University, China. We included participants aged older than 18 years, diagnosed with any of the prespecified immune-mediated liver disorders (AIH or AIH-PBC overlap syndrome) (22–24), no SARS-CoV-2 infection before receipt of the first vaccine dose (determined based on either a negative anti-SARS-CoV-2 IgM/IgG test or the absence of a positive polymerase chain reaction assay result for SARS-CoV-2, with no history of suspected clinical SARS-CoV-2 infection), completed whole-course COVID-19 vaccination (2 doses of BBIBP-CorV or CoronaVac vaccine), and were able to understand and complete questionnaires. Healthy participants were included as healthy controls (HCs). Participants with known pregnancy during study entry, those who did not complete the full course of vaccination, and those who provided incomplete vaccination information (including the date of first vaccine dose and complete vaccination and vaccine manufacturers) were excluded. Blood samples were collected for serological assays for SARS-CoV-2 at least 21 days after the whole-course vaccination from AILD patients and HCs.

This study was approved by the Ethics Committee of the Second Affiliated Hospital of Chongqing Medical University and in accordance with the ethical guidelines of the Declaration of Helsinki. Written informed consent was obtained from all participants. This study has been registered at ClinicalTrials.gov (NCT05007665).

### Variables and definitions

Clinical characteristics, including age, sex, body mass index (BMI), comorbidities, history of diseases and concomitant medications, of all patients were collected *via* a standardized questionnaire. The presence or absence of cirrhosis was confirmed using clinical or biochemical evidence, FibroScan, liver imaging (ultrasound, CT, or MRI) and endoscopy. The concomitant medications, especially types and doses of immunosuppression, were further confirmed through the prescribing information system in the hospital.

All adverse events (AEs) within 7 days and 30 days after COVID-19 vaccination were recorded and graded according to the National Medical Products Administration of China (version 2019). AEs related to vaccination were judged by investigators. Safety was evaluated by determining the overall incidence of AEs.

### SARS-CoV-2 antibody test

The SARS-CoV-2 antibody against the spike protein receptor-binding domain (anti-RBD-IgG) was detected by indirect ELISA using the SARS-CoV-2 RBD antibody detection kit (Sino Biological, Beijing, China). The lower limit of quantification is 5.0 arbitrary units per mL (AU/mL). The neutralizing antibodies (NAbs) were detected by the competitive ELISA method using the SARS-CoV-2 neutralizing antibody detection kit (Sino Biological, Beijing, China). The details were described in our previous study (25).

### SARS-CoV-2-specific B cells responses

Previous studies have described the selection of markers of memory B cells (MBCs) and their subsets (26, 27). For SARS-CoV-2-specific B cells detection, biotinylated SARS-CoV-2 spike RBD protein (Sino Biological, 40592-V08H2-B) was mixed with streptavidin BV421 (Biolegend, 405225) at a 4:1 molar ratio for one hour at 4°C to obtain the antigen probe. According to the manufacturer’s instructions, peripheral blood mononuclear cells (PBMCs) were isolated from heparinized whole blood by Histopaque (Sigma–Aldrich, 10771) density gradient centrifugation. After washing with FACS buffer (PBS+2% FBS), PBMCs were stained for 30 minutes at 4°C using an antigen probe (1:33.3) and the following conjugated antibodies: anti-human CD3 (300430, Biolegend, 1:50), anti-human CD19 (302212, Biolegend, 1:50), anti-human CD21 (354918, Biolegend, 1:50), anti-human CD27 (356406, Biolegend, 1:50), anti-human IgG Fc (410722, Biolegend, 1:50), and anti-human IgM (314524, Biolegend, 1:50). After staining, the cells were rewashed and resuspended in 200 µl FACS buffer. Samples were then evaluated by flow cytometry (Beckman Coulter, CytoFLEX) and analyzed using FlowJo (Treestar, 10.0.7r2).

### Statistical analysis

Data are presented as the median (interquartile range, IQR) for continuous variables and proportions for categorical variables. Continuous variables were compared using Student’s t test for the variables of age and BMI. Categorical variables were compared using Fisher’s exact test or the chi-square test for sex, cirrhosis, comorbidities, immunosuppressants, and vaccine types. One-way analysis of variance (ANOVA) was used to compare the results of multiple groups, and Tukey’s correction was used to correct for comparisons between groups. Negative responses and relatively low antibody levels following SARS-CoV-2 vaccination were classified as poor antibody responses in previous studies (19, 28, 29). In this study, a poor antibody response was defined as an anti-RBD-IgG titer less than the median value (≤ 34.0 AU/ml) or negative for NAbs. Multiple logistic regression was used to explore the independent variables associated with poor antibody responses and presented as odds ratios (ORs) (95% confidence intervals, CIs) with adjustment for potential confounding factors. Statistical analysis was performed using EmpowerStats (http://www.empowerstats.com, X&Y Solutions, Inc., Boston, MA, USA) and R (http://www.Rproject.org, the R Foundation). All statistical tests were two-sided, and p < 0.05 was considered statistically significant.

## Results

### Characteristics of participants

76 eligible AILD patients and 136 HCs were included in the study. The characteristics of the participants are shown in **Table 1**. Briefly, the median age was 54.0 years (IQR: 48.8-60.2 years) in AILD patients and 52.0 years (IQR: 33.0-62.2 years) in the HC group. The majority of participants were female (85.5% [65/76] in AILD patients and 55.1% [75/136] in HC). The median BMI and proportion of vaccine types in AILD patients and HCs were similar (22.5 kg/m^2^ [IQR: 21.2-23.9 kg/m^2^] vs. 22.9 kg/m^2^ [IQR: 21.1-25.3 kg/m^2^]). The median postvaccination time was 41.5 days (IQR: 29.0-66.2 days) and 55.0 (IQR: 33.0-86.5 days) for the AILD patients and HCs, respectively. Additionally, among these AILD patients, 20 (26.3%) had cirrhosis, and almost half (46.1%, 35/76) received one or more immunosuppressant medications.

**Table 1:** Characteristics of the Participants.

Variables	AILD(n=76)	Healthy controls(n=136)
Age, years	54.0 (48.8-60.2)	52.0 (33.0-62.2)
<55	39 (51.3)	78 (57.4)
≥55	37 (48.7)	58 (42.6)
Sex		
Female	65 (85.5)	75 (55.1)
Male	11 (14.5)	61 (44.9)
BMI, kg/m^2^	22.5 (21.2-23.9)	22.9 (21.1-25.3)
<24	57 (75.0)	83 (61.0)
≥24	19 (25.0)	53 (39.0)
Cirrhosis		
Yes	20 (26.3)	0 (0)
No	56 (73.7)	0 (0)
Comorbidities		
NAFLD	9 (11.8)	0 (0)
Alcoholic liver disease	2 (2.6)	0 (0)
Chronic hepatitis B	2 (2.6)	0 (0)
Diabetes	10 (13.2)	0 (0)
Hypertension	12 (15.8)	0 (0)
Tumor^†^	9 (11.8)	0 (0)
Other autoimmune diseases^‡^	15 (19.7)	0 (0)
Vaccine		
BBIBP-CorV	21 (27.6)	56 (41.2)
CoronaVac	49 (64.5)	72 (52.9)
BBIBP-CorV & CoronaVac	6 (7.9)	8 (5.9)
Immunosuppressant medication		
Prednisone	27 (35.5)	0 (0)
Azathioprine	17 (22.4)	0 (0)
Mycophenolate mofetil	3 (3.9)	0 (0)
Other medications^§^	10 (13.2)	0 (0)
Days between final dose and antibody test	41.5 (29.0-66.2)	55.0 (33.0-86.5)

Data are presented as n (%) or median (IQR). ^†^These include liver cancer, rectal cancer, thyroid cancer, myoma of the uterus, and esophageal leiomyoma. ^‡^These include Hashimoto’s thyroiditis, Sjogren’s syndrome, and polymyositis. ^§^These include methotrexate, tacrolimus, hydroxychloroquine and total glycoside of paeony. AILD, autoimmune liver diseases; BMI, body mass index; NAFLD, non-alcoholic fatty liver disease.

### COVID−19 vaccination safety

The overall incidence of AEs within 7 days (25.0% [19/76] vs 17.6% [24/136]) and 30 days (25.0% [19/76] vs 17.6% [24/136]) after COVID-19 vaccination was slightly higher in AILD patients than in the HC group (p = 0.20) (**Table 2**). All AEs were mild, and none of them had any serious AEs. The common AEs in AILD patients in local AEs were pain at the injection site (4.0%, 3/76); in systemic AEs were fatigue (5.3%, 4/76) and headache (5.3%, 4/76). The most common AE in HC was local pain at the injection site (8.1%, 11/136) (**Table 2**). Notably, one patient increased the serum gamma-glutamyl-transpeptidase (GGT) level from 20 U/L to 90 U/L (upper limit of normal: 45 U/L) after vaccination and returned to normal after continuing the original treatment strategy during the follow-up. Another patient’s antinuclear antibody (ANA) was positive at a titer of 1:320 before vaccination. It increased to 1:1000 after vaccination, but the liver function test, serum IgG, anti-liver-kidney microsomal, anti-smooth muscle, anti-mitochondrial antibodies, and anti-soluble liver antigen were normal, and the patient had no symptoms of discomfort.

**Table 2:** Adverse events of COVID-19 vaccination in participants.

Variables	AILD(n=76)	Healthy controls (n=136)	*p*-value
Overall adverse events within 7 days	19 (25.0)	24 (17.6)	0.20
Overall adverse events within 30 days	19 (25.0)	24 (17.6)	0.20
Local adverse events			
Pain	3 (4.0)	11 (8.1)	0.38
Itch	0 (0)	1 (0.7)	1.00
Redness	0 (0)	2 (1.5)	0.54
Swelling	2 (2.6)	4 (2.9)	1.00
Systemic adverse events			
Drowsiness	0 (0)	3 (2.2)	0.55
Shoulder pain	0 (0)	1 (0.7)	1.00
Fatigue	4 (5.3)	1 (0.7)	0.11
Numbness of limb	1 (1.3)	1 (0.7)	1.00
Nausea	1 (1.3)	0 (0)	0.36
Lower extremity edema	1 (1.3)	0 (0)	0.36
Headache	4 (5.3)	0 (0)	0.02
Myalgia	2 (2.6)	0 (0)	0.13
Elevated liver enzymes	1 (1.3)	0 (0)	0.36

Data are presented as n (%).

### Antibody responses after COVID-19 vaccination

**Figure 1:**
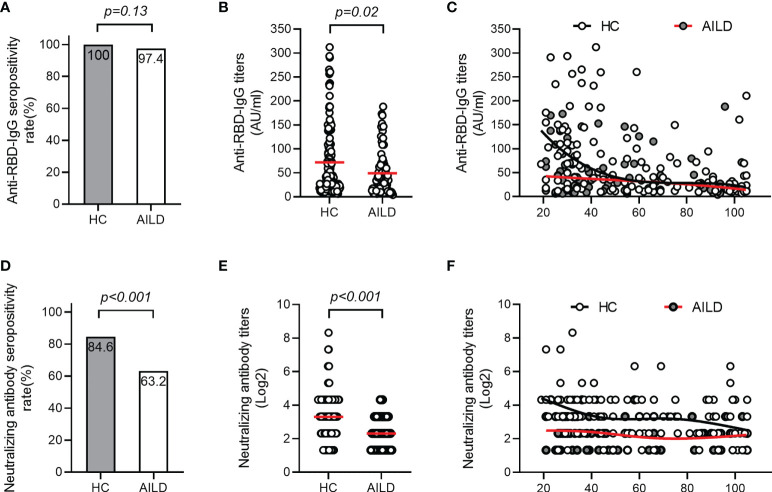
Antibody responses after COVID-19 vaccination in patients with AILD and healthy controls. The seropositivity rates and titers of anti-RBD-IgG **(A, B)** and NAbs **(D, E)** in patients with AILD and healthy controls. The distribution of anti-RBD-IgG **(C)** and NAbs **(F)** antibody titers over time in patients with AILD and healthy controls. AILD, autoimmune liver disease; anti-RBD-IgG, spike receptor-binding domain IgG antibody; NAbs, neutralizing antibodies.

The seropositivity for anti-RBD-IgG was 97.4% (74/76) in AILD patients, which was similar to that in the HC group (100%) (p = 0.13) (**Figure 1A**). However, anti-RBD-IgG levels were significantly lower in AILD patients than those in HCs (mean: 49.1 AU/mL vs 71.9 AU/mL, p = 0.02) (**Figure 1B**). Regarding NAbs, seropositivity (63.2% [48/76] vs 84.6% [115/136]) and antibody titers were both significantly lower in AILD patients than those in HCs (p < 0.001) (**Figures 1D, E**). Compared with controls, anti-RBD-IgG and NAbs levels seemed to decrease slightly over time after the second dose vaccination in AILD patients (**Figures 1C, F**).

### Effect of immunosuppressants on antibody responses

**Figure 2:**
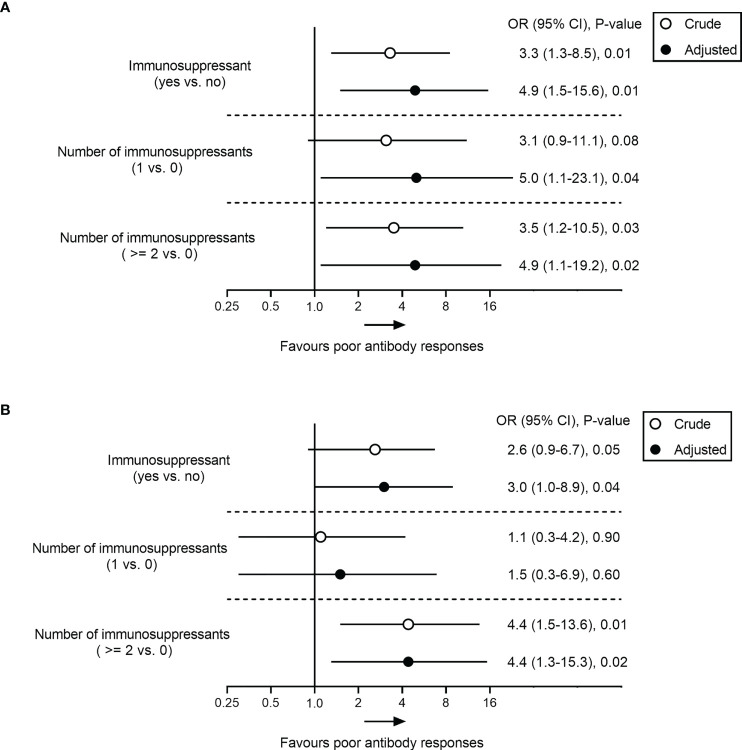
Association analysis between immunosuppressant and poor antibody responses to SARS-CoV-2 vaccine in patients with AILD. Odds ratio (95% CI) of poor response to RBD-IgG **(A)** and NAbs **(B)** in immunosuppressant-treated AILD patients in crude (unadjusted) and adjusted models compared with healthy controls. The adjusted model was adjusted age, BMI, sex, cirrhosis, comorbidities, types of vaccine, and days between final dose and antibody test. AILD, autoimmune liver diseases; BMI, body mass index; CI, confidence interval; OR, odds ratio; SARS-CoV-2, severe acute respiratory syndrome coronavirus 2.

Analysis of clinical features showed no significant correlations between poor antibody responses and parameters such as age, sex, BMI, cirrhosis, and comorbidities (all p > 0.05) (**Table 3**). However, low-level antibodies were significantly related to the types of vaccine (p = 0.01) (**Table 3**, **Supplementary Figure 1A**) and the use of immunosuppressants (p = 0.04) (**Table 3**; **Supplementary Figure 1C**), and these results were consistent with univariate analysis (**Supplementary Table 1**). Furthermore, after adjusting for potential confounding factors (age, BMI, sex, cirrhosis, comorbidities, types of vaccine, and days between final dose and antibody test) in multiple logistic regression analysis, the use of immunosuppressants remained significantly related to low-level antibody levels (**Figure 2**). Compared with patients with no immunosuppressive medication, the crude odds ratio (OR) of low-level antibody response risk among patients who used immunosuppressants was 3.3 (95% CI, 1.3-8.5; p = 0.01), and their adjusted OR (aOR) increased to 4.9 (95% CI, 1.5-15.6; p = 0.01). Notably, the risk trend does not seem to increase with the number of immunosuppressants. The aORs of the use of one and more immunosuppressive medications were 5.0 (95% CI, 1.1-23.1; p = 0.04) and 4.9 (95% CI, 1.1-19.2; p = 0.03), respectively (**Figure 2**).

**Table 3:** Distribution of clinical characteristics by serum antibody titers to SARS-CoV-2 vaccine in patients with AILD.

Variables	Anti-RBD-IgG		*p*-value	NAbs		*p*-value
	Low-level group^*^ (n=38)	High-level group^*^ (n=38)		Negative (n=28)	Positive (n=48)	
Age, years	55.0 (52.0-58.8)	54.5 (47.5-62.2)	0.72	55.0 (48.8-60.2)	54.5 (49.8-60.5)	0.99
<55	18 (47.4)	19 (50.0)	0.82	13 (46.4)	24 (50.0)	0.76
≥55	20 (52.6)	19 (50.0)		15 (53.6)	24 (50.0)	
Sex						
Female	34 (89.5)	31 (81.6)	0.33	24 (85.7)	41 (85.4)	0.97
Male	4 (10.5)	7 (18.4)		4 (14.3)	7 (14.6)	
BMI, kg/m^2^	22.5 (21.2-24.1)	22.5 (21.0-23.8)	1.00	22.9 (21.2-24.1)	22.4 (21.2-23.8)	0.71
<24	27 (71.1)	30 (78.9)	0.43	20 (71.4)	37 (77.1)	0.58
≥24	11 (28.9)	8 (21.1)		8 (28.6)	11 (22.9)	
Cirrhosis	9 (23.7)	11 (28.9)	0.60	7 (25.0)	13 (27.1)	0.84
Comorbidities						
0	16 (42.1)	16 (42.1)	1.00	10 (35.7)	22 (45.8)	0.39
≥1	22 (57.9)	22 (57.9)		18 (64.3)	26 (54.2)	
Vaccine						
BBIBP-CorV	16 (42.1)	5 (13.2)	0.01	13 (46.4)	8 (16.7)	0.01
CoronaVac	21 (55.3)	28 (73.7)		15 (53.6)	34 (70.8)	
BBIBP-CorV & CoronaVac	1 (2.6)	5 (13.2)		0 (0)	6 (12.5)	
Immunosuppressant						
0	15 (39.5)	26 (68.4)	0.04	11 (39.3)	30 (62.5)	0.02
1	9 (23.7)	5 (13.2)		4 (14.3)	10 (20.8)	
≥2	14 (36.8)	7 (18.4)		13 (46.4)	8 (16.7)	

Data are presented as n (%) or median (IQR). ^*^Stratified by the median level of anti-RBD-IgG to SARS-CoV-2 vaccine. AILD, autoimmune liver disease; BMI, body mass index; IQR, interquartile range; SARS-CoV-2, severe acute respiratory syndrome coronavirus 2

Similar results were also observed for NAbs responses in the AILD patients. Negative NAbs were significantly associated with the types of vaccine (p = 0.01) (**Supplementary Figure 1B**) and immunosuppressants (p = 0.02) (**Supplementary Figure 1D**), except for age, sex, BMI, cirrhosis, and comorbidities (**Table 3**) (**Supplementary Table 1**). After adjusting for confounding factors, immunosuppressants were associated with a reduced probability of NAbs seropositivity (aOR, 3.0; 95% CI, 1.0-8.9; p = 0.04), especially when ≥2 immunosuppressive medications were used (aOR, 4.4; 95% CI, 1.3-15.3; p = 0.02) (**Figure 2**).

Concisely, the results suggested that immunosuppressive therapy was an independent risk factor for poor antibody responses to COVID-19 vaccination in patients with AILD.

### Specific B cells responses after COVID-19 vaccination

**Figure 3:**
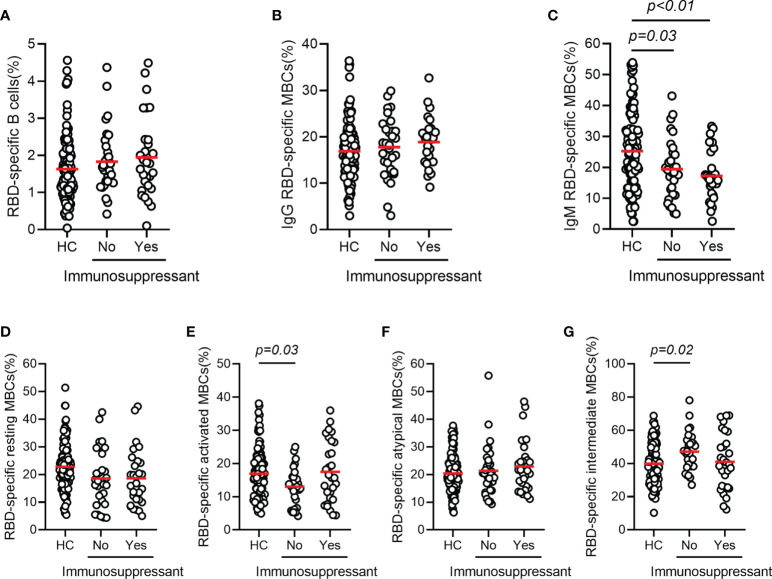
RBD-specific B cell responses after COVID-19 vaccination in patients with AILD and healthy controls. Frequency of RBD-specific B cells **(A)**, IgG RBD-specific MBCs **(B)**, IgM RBD-specific MBCs **(C)**, RBD-specific resting MBCs **(D)**, RBD-specific activated MBCs **(E)**, RBD-specific atypical MBCs **(F)** and RBD-specific intermediate MBCs **(G)** in patients with AILD and healthy controls. AILD, autoimmune liver disease; MBCs, memory B cells; RBD, receptor-binding domain.

The mean frequencies of B cells (CD3^-^CD19^+^) in AILD patients with and without immunosuppressive therapy were 9.42% and 8.63%, respectively, with no statistically significant difference compared with those in HCs (8.82%). The frequency of total MBCs (CD3^-^CD19^+^CD27^+^) was significantly higher in HC group than in patients with AILD who were not treated with immunosuppressants (37.6% vs 29.7%, p = 0.01), while the frequency in patients treated with immunosuppressants was at an intermediate level (**Supplementary Figure 2**). To further investigate the humoral immune response to the SARS-CoV-2 vaccine, the frequency and phenotype of specific B cells were also detected. As expected, the percentage of specific B cells was very low in the peripheral blood of AILD patients and the HC group. No significant difference was found in the frequency of RBD-specific B cells (CD3^-^CD19^+^RBD^+^) and IgG RBD-specific memory B cells (IgG^+^CD3^-^CD19^+^RBD^+^CD27^+^) between the AILD and HC groups, regardless of immunosuppressants (**Figures 3A, B**). However, the frequency of IgM RBD-specific MBCs (IgM^+^CD3^-^CD19^+^RBD^+^CD27^+^) was significantly lower in AILD patients with (17.2% vs 25.3%, adjusted p < 0.01) or without immunosuppressants (19.4% vs 25.3%, adjusted p = 0.03) than in the HC group (**Figure 3C**). To better understand the functional phenotype of RBD-specific MBCs, we further compared RBD-specific resting MBCs (CD3^-^CD19^+^RBD^+^CD21^+^CD27^+^), RBD-specific activated MBCs (CD3^-^CD19^+^RBD^+^CD21^-^CD27^+^), RBD-specific atypical MBCs (CD3^-^D19^+^RBD^+^CD21^-^CD27^-^), and RBD-specific intermediate MBCs (CD3^-^CD19^+^RBD^+^CD21^+^CD27^-^) between AILD patients and HC groups. Compared with HCs, AILD patients without immunosuppressants had a lower frequency of RBD-specific activated MBCs (13.0% vs 16.9%, adjusted p = 0.03) and a higher frequency of RBD-specific intermediate MBCs (47.1% vs 39.9%, adjusted p = 0.02), but not in patients with immunosuppressants. Moreover, there was no significant difference in RBD-specific resting MBCs and RBD-specific atypical MBCs between AILD patients and the HC groups (**Figures 3D–G**). These results indicate that patients with AILD may develop humoral immunity as robust as in a healthy population when receiving a booster dose or against SARS-CoV-2 infection despite ongoing immunosuppression.

## Discussion

AILD is a chronic disease characterized by immune-mediated disorders. Safety and immunogenicity have been of concern in patients with AILD since vaccination against COVID-19. In this prospective observational study, we found that inactivated SARS-CoV-2 vaccines achieved a favorable safety profile, but their immunogenicity is compromised in patients with AILD. The use of immunosuppressants had an estimated 3- to 5-fold increased risk of poor antibody responses to the SARS-CoV-2 vaccine. Moreover, the specific MBCs responses were comparable between patients in the AILD and HC groups despite ongoing immunosuppression.

Similar to the general population and other chronic liver diseases, such as NAFLD, liver transplantation, chronic hepatitis B, and liver cirrhosis, adverse events related to the COVID-19 vaccine in patients with AILD were mild and self-resolved within a few days after vaccination (19, 30–32). However, after the whole-course vaccination, one patient experienced an increased serum GGT level, and another experienced a sharply increased ANA titer. Because they refused liver biopsy, it is unclear whether this phenomenon reflects the fluctuation of the disease itself or whether the vaccine induces new-onset autoimmune diseases. Fortunately, we did not observe any evidence of clinical deterioration in neither patients during follow-up over 6 months. Herein, we believe that the COVID-19 inactivated vaccine is safe in patients with AILD.

Among the AILD patients, the COVID-19 inactivated vaccine showed an efficient antibody response of anti-RBD-IgG (97.4%). This was similar in a multicenter study of NAFLD patients in China (95.5%) but much higher than the reported seropositivity of SARS-CoV-2 RBD-specific antibodies in patients with chronic hepatitis B virus infection who were also vaccinated with inactivated COVID-19 vaccines (87.25%) (31, 32). This can be attributed to the large percentage of patients in our study were female (85.5% vs 27.5%). Xiang et al. found that female patients exhibited higher seropositivity for SARS-CoV-2 RBD-specific antibodies than males with chronic hepatitis B (95.1% vs 84.3%) ^32^. A similar finding that female vaccine recipients showed more robust antibody responses to COVID-19 vaccination was also reported in a clinical trial in Turkey (30). Similar to chronic hepatitis B, the NAbs seropositivity was lower than that of anti-RBD-IgG in patients with AILD. We found that immunosuppressants have an estimated 3- to 5-fold increased risk of poor antibody responses to the SARS-CoV-2 vaccine. In a nationwide multicenter prospective cohort study of 125 patients with multiple sclerosis, Bsteh *et al.* reported that immunosuppressive therapy could significantly reduce the probability of NAbs seropositivity after symptomatic COVID-19 (OR, 0.51; 95% CI, 0.17-0.82) (33). This finding might partly explain why the seropositivity and titer of NAbs in AILD patients are lower than those in HCs. However, a cohort study in patients with immune-mediated inflammatory disorders on immunosuppressants showed that only certain specific immunosuppressants attenuated the humoral responses after SARS-CoV-2 mRNA vaccine (21). More studies are needed to compare the effects of multiple types of immunosuppressants on different types of SARS-CoV-2 vaccines.

This study found that the RBD-specific MBCs responses were comparable between patients with AILD and HCs despite ongoing immunosuppression. It’s consistent with Kirchner et al. in a small study showing that patients with AIH receiving immunosuppressive therapy still developed strong humoral and cellular immunity to SARS-CoV-2 (34). Given the role of MBCs, it is speculated that patients with AILD may develop humoral immunity as robust as those in a healthy population when receiving a booster dose or against SARS-CoV-2 infection.

There are several main limitations in this study. First, a lack of longitudinal serial antibody testing limits the possibility of measuring a change in antibody levels individually. Second, the relatively small sample size of the study and the lack of subgroup analyses, such as strength of immunosuppression and antibody response, may reduce the reliability and limit the generalizability of the findings. Third, the antibody response is only part of the immunogenicity of the COVID-19 vaccine, so there is a need to explore the T-cell response. However, given the unprecedented nature of the COVID-19 pandemic and the low prevalence of AILD, we believe that our study offers valuable insights into the management of these patients to clinicians.

In conclusion, the COVID-19 inactivated SARS-CoV-2 vaccines (BBIBP-CorV and CoronaVac) are safe, but their immunogenicity is compromised in patients with AILD. In addition, immunosuppressants are significantly associated with poor antibody responses to the SARS-CoV-2 vaccines. These results could inform physicians and policymakers about decisions on screening the populations at higher risk of poor antibody responses to SARS-CoV-2 vaccines and providing additional vaccinations in patients with AILD.

## Data availability statement

The original contributions presented in the study are included in the article/**Supplementary Material**. Further inquiries can be directed to the corresponding authors.

## Ethics statement

The studies involving human participants were reviewed and approved by ethics committee of the Second Affiliated Hospital of Chongqing Medical University. The patients/participants provided their written informed consent to participate in this study.

## Author contributions

The authors DC, DZ, and HR contributed to the conception and design and critical revision of important intellectual content. Data collection was performed by YW, LA, MK, ZC, MC, MP, NL, and PH. Statistical analysis was performed by HL and YW. The first draft of the manuscript was written by HL. All authors approved the final version and agreed to be accountable for all aspects of the work.

## Funding

This work is supported by the National Science and Technology Major Project of China (2017ZX10202203-007, 2017ZX10202203-008, 2018ZX10302206-003) and a pilot project of clinical cooperation between traditional Chinese and western medicine for significant and complicated diseases of the National Administration of Traditional Chinese Medicine: hepatic fibrosis.

## Acknowledgments

We also acknowledge the support of the Kuanren Talents Program of the Second Affiliated Hospital of Chongqing Medical University, the National Natural Science Foundation of China (81772198,81902068), and the Natural Science Foundation of Chongqing, China (cstc2020jcyj-msxmX0389).

## Conflict of interest

The authors declare that the research was conducted in the absence of any commercial or financial relationships that could be construed as a potential conflict of interest.

## Publisher’s note

All claims expressed in this article are solely those of the authors and do not necessarily represent those of their affiliated organizations, or those of the publisher, the editors and the reviewers. Any product that may be evaluated in this article, or claim that may be made by its manufacturer, is not guaranteed or endorsed by the publisher.

## Supplementary material

Supplementary Figure 1 Antibody response rates after COVID-19 vaccination. The seropositivity rates (A-B) and titers (C-D) of anti-RBD-IgG and NAbs among the different types of vaccines (BBIBP-CorV, CoronaVac and BBIBP-CorV & CoronaVac) or different groups of immunosuppressive medications (0, 1, ≥2). AILD, autoimmune liver diseases; anti-RBD-IgG, spike receptor-binding domain IgG antibody; NAbs, neutralizing antibodies.

Supplementary Figure 2 The frequencies of total B cells and MBCs in AILD patients with and without immunosuppressive therapy. The frequencies of B cells (CD3-CD19+) **(A)** and MBCs (CD3-CD19+CD27+) in AILD patients after COVID-19 vaccination. AILD, autoimmune liver diseases; COVID-19, coronavirus disease 2019; HCs, healthy controls; MBCs, memory B cells.

Supplementary Table 1 Univariate analysis of factors associated with poor antibody responses to SARS-CoV-2 vaccine in patients with AILD.

## Abbreviations

AEs, adverse events; AIH, autoimmune hepatitis; AILD, autoimmune liver diseases; ANA, antinuclear antibody; BMI, body mass index; CI, confidence interval; COVID-19, coronavirus disease 2019; GGT, gamma-glutamyl-transpeptidase; HCs, healthy controls; IgG, immunoglobulin G; IQR, interquartile range; MBCs, memory B cells; NAbs, neutralizing antibodies; NAFLD, non-alcoholic fatty liver disease; OR, odds ratio; PBC, primary biliary cholangitis; RBD, receptor-binding domain; SARS-CoV-2, severe acute respiratory syndrome coronavirus 2.
