# Supplementary figures and images for: Association between immunosuppressants and poor antibody responses to SARS-CoV-2 vaccines in patients with autoimmune liver diseases
Source: Front Immunol. 2022 Oct 5;13:988004. doi: 10.3389/fimmu.2022.988004 (PMC9579272; doi:10.3389/fimmu.2022.988004)

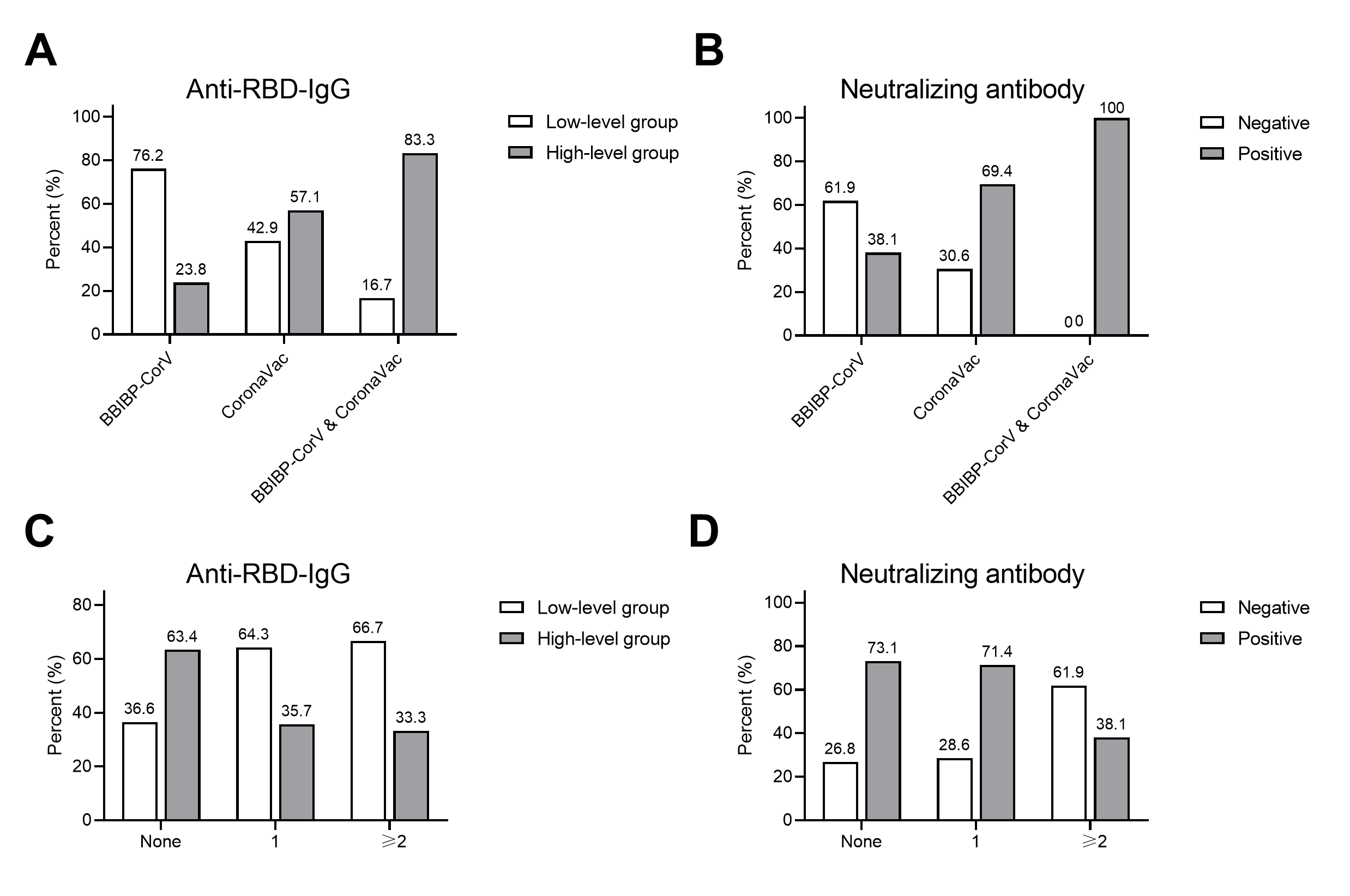

Supplement: Supplementary Figure 1 — Antibody response rates after COVID-19 vaccination. The seropositivity rates (A-B) and titers (C-D) of anti-RBD-IgG and NAbs among the different types of vaccines (BBIBP-CorV, CoronaVac and BBIBP-CorV & CoronaVac) or different groups of immunosuppressive medications (0, 1, ≥2). AILD, autoimmune liver diseases; anti-RBD-IgG, spike receptor-binding domain IgG antibody; NAbs, neutralizing antibodies. [file Image_1.tif]

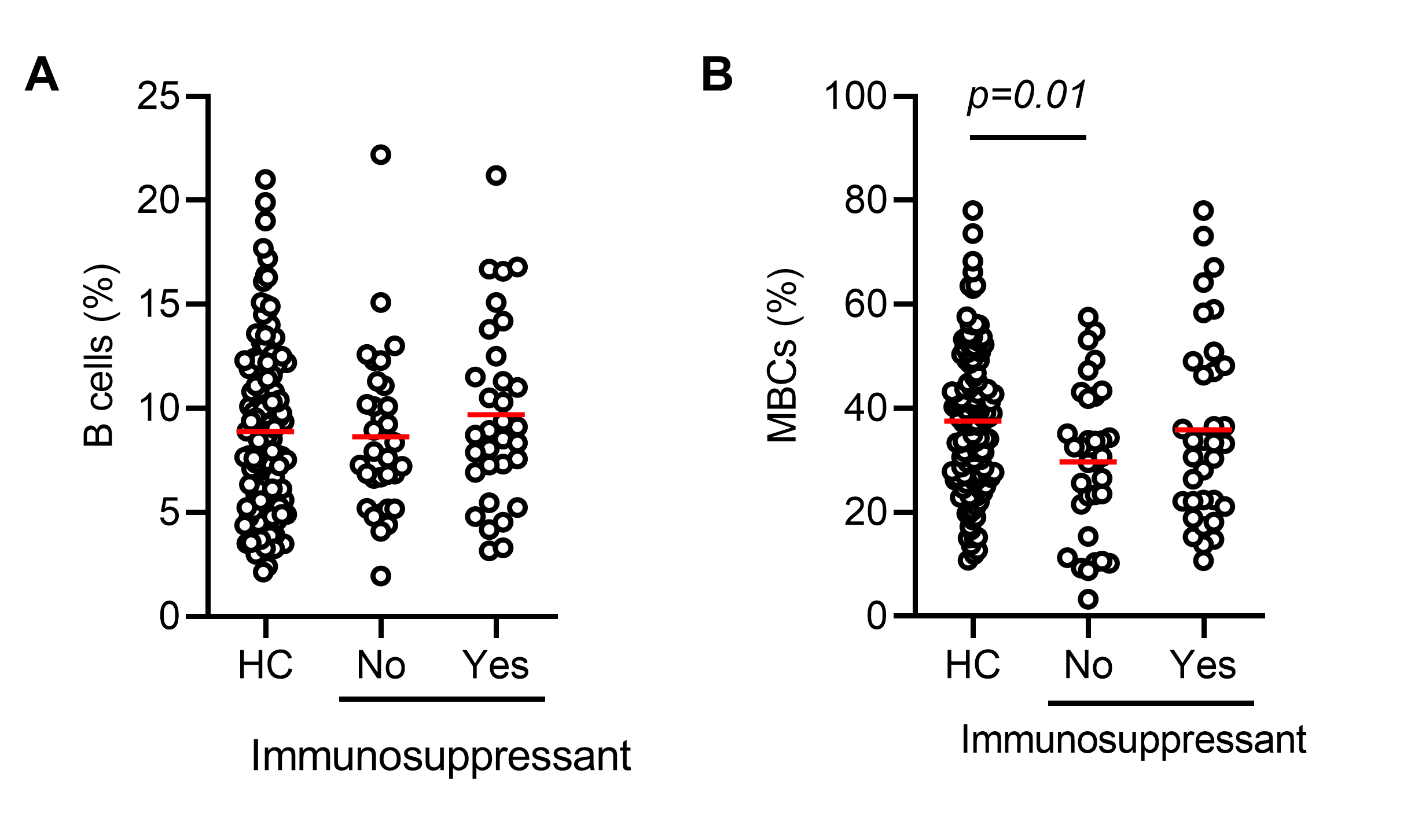

Supplement: Supplementary Figure 2 — The frequencies of total B cells and MBCs in AILD patients with and without immunosuppressive therapy. The frequencies of B cells (CD3-CD19+) (A) and MBCs (CD3-CD19+CD27+) in AILD patients after COVID-19 vaccination. AILD, autoimmune liver diseases; COVID-19, coronavirus disease 2019; HCs, healthy controls; MBCs, memory B cells. [file Image_2.tif]
