# Supplementary material for: Association between immunosuppressants and poor antibody responses to SARS-CoV-2 vaccines in patients with autoimmune liver diseases
Source: Front Immunol. 2022 Oct 5;13:988004. doi: 10.3389/fimmu.2022.988004 (PMC9579272; doi:10.3389/fimmu.2022.988004)
Supplement: Supplementary Table 1 — Univariate analysis of factors associated with poor antibody responses to SARS-CoV-2 vaccine in patients with AILD. [file Table_1.docx]

**Supplementary Table 1. Univariate analysis of factors associated with poor antibody responses to SARS-CoV-2 vaccine in patients with AILD.**

| Variables | Anti-RBD-IgG | NAbs |
| --- | --- | --- |
| Age, years | 1.0 (1.0-1.1) 0.60 | 1.0 (0.9-1.1) 0.99 |
| Sex |  |  |
| Male | 1.0 | 1.0 |
| Female | 1.9 (0.5-7.2) 0.33 | 1.0 (0.3-3.9) 0.97 |
| BMI, kg/m^2^ | 1.0 (0.8-1.2) 0.99 | 1.0 (0.9-1.2) 0.71 |
| Cirrhosis |  |  |
| 0 | 1.0 | 1.0 |
| 1 | 0.8 (0.3-2.1) 0.60 | 0.9 (0.3-2.6) 0.84 |
| Comorbidities |  |  |
| 0 | 1.0 | 1.0 |
| 1 | 1.0 (0.4-2.5) 1.00 | 1.5 (0.6-4.0) 0.39 |
| Vaccine |  |  |
| BBIBP-CorV | 1.0 | 1.0 |
| CoronaVac | 0.2 (0.1-0.7) 0.01 | 0.3 (0.1-0.8) 0.02 |
| BBIBP-CorV & CoronaVac | 0.1 (0.0-0.7) 0.02 | 0.0 (0.0-Inf.) 0.99 |
| Immunosuppressant |  |  |
| No | 1.0 | 1.0 |
| Yes | 3.3 (1.3-8.5) 0.01 | 2.6 (1.0-6.7) 0.05 |

Data are presented as OR (95% CI), and p-values. AILD, autoimmune liver disease; BMI, body mass index; CI, confidence interval; Inf, infinite; NAbs, neutralizing antibodies; OR, odds ratio; RBD, receptor-binding domain; SARS-CoV-2, severe acute respiratory syndrome coronavirus 2.
